# Substructure imaging of heterogeneous nanomaterials with enhanced refractive index contrast by using a functionalized tip in photoinduced force microscopy

**DOI:** 10.1038/s41377-018-0069-y

**Published:** 2018-10-10

**Authors:** Junghoon Jahng, Heejae Yang, Eun Seong Lee

**Affiliations:** 1 Center for Nanocharacterization, Korea Research Institute of Standards and Science (KRISS), Daejeon, 34113 Republic of Korea; 2 Department of Materials Engineering, Advanced Fibrous Materials Laboratory, University of British Columbia, Vancouver, BC V6T 1Z4 Canada

## Abstract

The opto-mechanical force response from light-illuminated nanoscale materials has been exploited in many tip-based imaging applications to characterize various heterogeneous nanostructures. Such a force can have two origins: thermal expansion and induced dipoles. The thermal expansion reflects the absorption of the material, which enables one to chemically characterize a material at the absorption resonance. The induced dipole interaction reflects the local refractive indices of the material underneath the tip, which is useful to characterize a material in the spectral region where no absorption resonance occurs, as in the infrared (IR)-inactive region. Unfortunately, the dipole force is relatively small, and the contrast is rarely discernible for most organic materials and biomaterials, which only show a small difference in refractive indices for their components. In this letter, we demonstrate that refractive index contrast can be greatly enhanced with the assistance of a functionalized tip. With the enhanced contrast, we can visualize the substructure of heterogeneous biomaterials, such as a polyacrylonitrile-nanocrystalline cellulose (PAN-NCC) nanofiber. From substructural visualization, we address the issue of the tensile strength of PAN-NCC fibers fabricated by several different mixing methods. Our understanding from the present study will open up a new opportunity to provide enhanced sensitivity for substructure mapping of nanobiomaterials, as well as local field mapping of photonic devices, such as surface polaritons on semiconductors, metals and van der Waals materials.

## Photoinduced force microscopy: Enhanced sensitivity

Contamination of a scientific instrument is usually considered to be an undesirable outcome. However, researchers from Korea and Canada have now discovered that it can actually benefit an imaging technique called photoinduced force microscopy (PiFM). PiFM is a high-resolution scanning technique that is capable of mapping local refractive index contrast of a sample by measuring the light-induced force that arises between the sample and a closely-spaced tip. Junghoon Jang and coworkers from the Korea Research Institute of Standards and Science (KRISS) and the University of British Columbia have now discovered that the presence of a small amount of polydimethylsiloxane (PDMS) contaminant on the tip can serve to functionalize it and significantly increase the strength of the sample-tip interaction. The team has shown that the functionalized tip can map the substructure of nanofibres of a biomaterial called polyacrylonitrile-nanocrystalline cellulose.

## Introduction

Characterizing and visualizing the heterogeneous compositions of various nanobiomaterials together with their nanoscale morphology is an essential part of a variety of disciplines in the fields of nanoscience and nanotechnology^1^. The optical response is indicative of the heterogeneous composition of materials because the complex index of refraction directly corresponds to the electronic/vibrational modes. Conventional optical microscopy and spectroscopy are powerful tools that enable one to probe ensembles of optical characteristics for heterogeneous materials^2,3^. However, the spatial resolution for conventional optical microscopy is limited by the optical diffraction to half the wavelength, which corresponds to several hundreds of nanometers to micrometers in the visible to infrared spectral range. Moreover, the spectrum suffers from inhomogeneous line broadening due to ensemble averaging within a large sampling volume.

The scan-probe technique, including optical near-field microscopy, is one of the popular techniques used to overcome the diffraction limit. Scattering type scanning near-field optical microscopy (s-SNOM) is a prime example^4^. However, it is still challenging to isolate the actual near-field response from the background, which requires complex methods for background suppression, often resulting in signal degradation^5,6^. An alternative nanoscale optical method combined with scanning probe microscopy is opto-mechanical force microscopy such as photoinduced force microscopy (PiFM)^7–10^, photothermal-induced resonance (PTIR) technique^11,12^, and peak-force infrared (PFIR) microscopy^13^, which utilizes the mechanical force exerted on a tip via the light-induced tip-sample interaction as a read-out mechanism. The light-induced forces in these techniques are localized within the tip radius, thereby enabling direct optical imaging of the sample with high spatial resolution. Among these microscopic techniques, PiFM can operate in noncontact/tapping mode to monitor the induced dipole force based on the dispersive refractive index^9^, as well as the thermal expansion force based on dissipative absorption^14^. The thermal force assists in the chemical characterization of heterogeneous materials at their absorption resonances, while the induced dipole force can image the local effective index distribution, which is related to the sample structure.

The tip quality plays a crucial role for successful imaging using tip-based scanning probe microscopy including PiFM. Unfortunately, it is known that most commercial tips suffer from contamination by exogenous materials following the manufacturing process^15,16^. The main contaminant is polydimethylsiloxane (PDMS), also known as silicone, which is derived from silicone oil used for shipping and packaging materials in commercial boxes. The lower-molecular-weight forms of this silicone polymer vaporize in the packaging box and eventually coat the cantilever as a thin uniform film during long packaging hours (>800 h)^15^. In most imaging applications, this contamination layer is negligible because the thickness is expected to be very thin, i.e., 1–2 nm^16^. However, since the film has a relatively high thermal expansion (907 × 10^−6^ K^−1^) and high extinction coefficient (*k* ~ 0.35  m^−1^) in the mid-infrared range, despite its small thickness of only a few nm, one might expect that the contaminant itself can induce a force via thermal expansion by absorbing the enhanced field near the tip apex^16^. Thus, the contaminant molecules can act as a sensitive sensor for a tip-enhanced field, which enables one to explore the substructure of compositional materials as the field depends on the effective index of refraction for the sample system.

In this letter, we first present unprecedented experimental evidence showing that a tiny amount of PDMS contaminant can generate a photoinduced force spectrum with high sensitivity. Next, we demonstrate that the PDMS contamination, which is considered a natural functionalization, can be utilized as a very sensitive sensor for the enhancement of local refractive index contrast to visualize the substructure of heterogeneous nano- and biomaterials in PiFM nanoscopy. Finally, by visualizing the substructure of an exemplar heterogeneous sample, such as a polyacrylonitrile-nanocrystalline cellulose (PAN-NCC) nanofiber, which shows a small difference in refractive index for the heterogeneous compositions, we address the issue of tensile strength for PAN-NCC fibers fabricated using several different mixing methods.

## Results

**Fig. 1:**
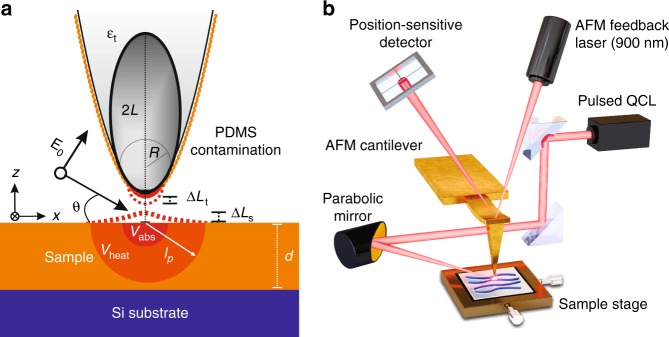
Schematics for tip-enhanced thermal expansion and the IR-PiFM system. **a** Diagram showing thermal expansion of the tip (Δ*L*_t_) and of the sample (Δ*L*_s_). The orange dashed layer on the tip is the PDMS contamination. The incident quantum cascade laser (QCL) illuminates the tip-sample junction at an angle of *θ*. *V*_abs_ is the absorption volume for the tip-enhanced field inside the sample. *V*_heat_ is the heated volume due to the absorbed heat over a single pulse width τ_*p*_. *d* is the thickness of the sample. **b** Sketch of the IR-PiFM experimental setup

When the narrow junction between a sharp metallic tip and a sample is illuminated by light, the electric field is tightly confined and highly enhanced near the tip end. If a material attached to the tip has an absorption resonance at the optical frequency, the material can absorb the enhanced field near the tip apex and dissipate heat. The consequent thermal expansion modulates the tip-sample gap distance (*H*), leading to a thermal expansion force via the van der Waals interaction, which can easily reach a few tens to a few hundreds of pN^14^, resulting in the generation of a force *gradient* signal in PiFM^17^. For a metallic tip with an organic material attached, light absorption followed by thermal expansion occurs at two locations: at the tip and at the sample, as sketched in Fig. 1a. If the material on the tip has no absorption near the sample resonance, the sample solely expands by an amount Δ*L*_s_ by absorbing the tip-enhanced field, which penetrates into the sample. Since the dipole force for organic molecules is typically one to two orders smaller than the thermal expansion force^14,18^, PiFM mapping for organic samples at their absorption resonance corresponds primarily to local absorption mapping for the sample. Conversely, if the sample has no absorption near the resonance of the organic material on the tip, the tip solely expands by Δ*L*_t_ by absorbing the enhanced field, the strength of which depends on the local refractive index of the sample. This implies that PiFM mapping corresponds to a dipole contrast (local refractive index contrast) for the sample via tip-enhanced thermal expansion of the organic material on the tip. Note that the photoinduced force spectrum at a certain position on the sample is still the absorption spectrum of the organic material on the tip. Lastly, if the absorption resonance of the organic material on the tip has the same frequency as that of the sample, the thermal force signal for the sample is convolved with the response of the tip because the tip-sample distance modulation is summed as Δ*L*_t_ + Δ*L*_s_.

**Fig. 2:**
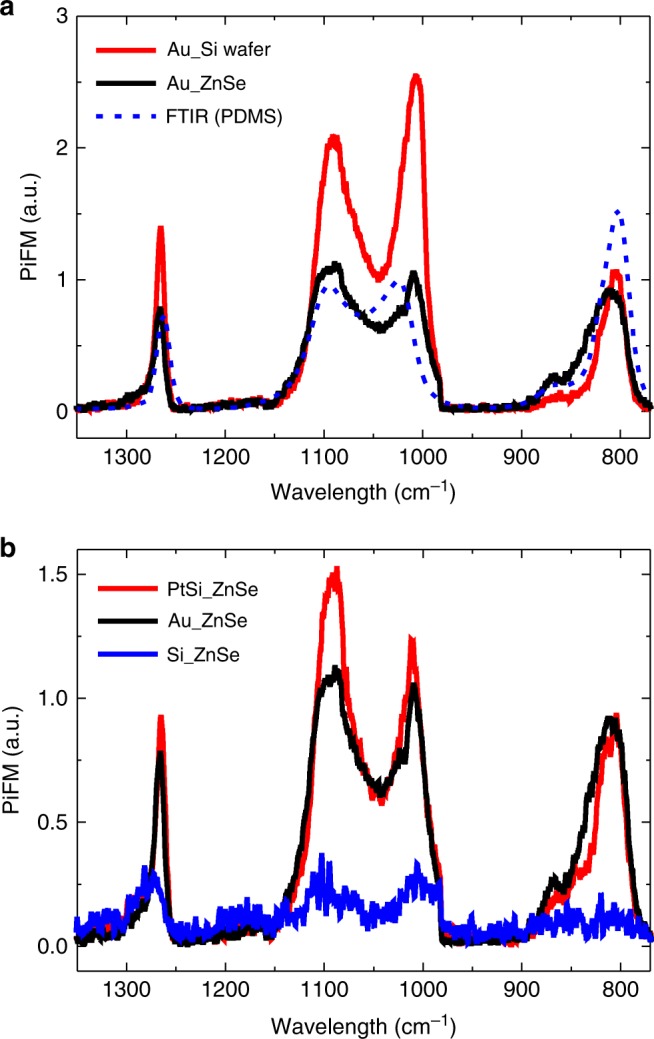
Measured PiFM spectra due to the PDMS contamination on a commercial tip. **a** PiFM spectra due to the thermal expansion of PDMS on a commercial Au-coated tip measured over a clean Si wafer (red solid line) and over a ZnSe window (black solid line). The blue dashed line shows the FTIR result for bulk PDMS obtained from the library^20^. **b** PiFM spectra due to tip expansion measured over a ZnSe window with respect to different tip-coating materials

We first examine the tip expansion behavior using the PDMS contamination layer on a commercial tip, which can be a very sensitive thermal probe for PiFM. Despite the small thickness of several nm for the contaminant layer, due to its high thermal expansion and high extinction coefficient in the mid-infrared range, the tip-enhanced thermal expansion easily reaches a few tens of pm, which results in several tens of pN via the van der Waals force [Section 2 of Supplementary Information]. In Fig. 2a, the PiFM spectra measured by an Au-coated tip with PDMS contaminant are plotted with respect to different substrate materials. The red solid line shows the data measured for a Si substrate whose index of refraction is 3.42 at 1268 cm^−1^ ^19^, and the black solid line shows data for a ZnSe substrate with an index of refraction of 2.41 at 1268 cm^−1^ ^20^. In the measured spectral range, no significant absorption is found for the Si wafer or ZnSe window except for the PDMS on the tip. The measured spectra correspond well to the FTIR spectrum obtained for PDMS (blue dashed line) from the library^20^. The peaks for PDMS (805, 1020, 1097, and 1268 cm^−1^) on the Si wafer are larger than those on the ZnSe window because of a larger enhanced field on the Si substrate due to the larger refractive index.

The field enhancement also depends on the radius of the tip and not just on the refractive index, due to the lightening-rod effect. In Fig. 2b, the measured PiFM spectra are plotted with respect to different commercial tips. The refractive indices for an Au-coated tip (PPP-NCHAu), PtSi-coated tip (PtSi-NCH), and bare-Si tip (PPP-NCHR) from *Nanosensors Inc*. are *n* ~ 7.70^21^, 8.27^22^, and 3.42^19^, respectively, at 1268 cm^−1^, with their tip radii guaranteed to be ~30, 25, and 10 nm, respectively. Note that the bulk refractive indices are still applicable until the coating thickness exceeds ten nm^23,24^. We can assume that the thickness of the PDMS layer is 1–2 nm for all the tips in this experiment because the three tip materials have a similar hydrophobicity^16,25,26^. Since the PtSi-coated tip has the highest refractive index and a relatively small tip radius compared to the other tips, the PiFM spectrum measured with a PtSi-coated tip shows the strongest signal, as expected. Note that even though the refractive index of the bare-Si tip is far smaller than the other metallic tips, an appreciable signal is still measured due to the small tip radius. Although the refractive index difference between the two metals (Au and PtSi) is relatively small, the measured signals show a clear difference because the tip radius of the Au-coated tip is relatively larger than that of the PtSi-coated tip.

**Fig. 3:**
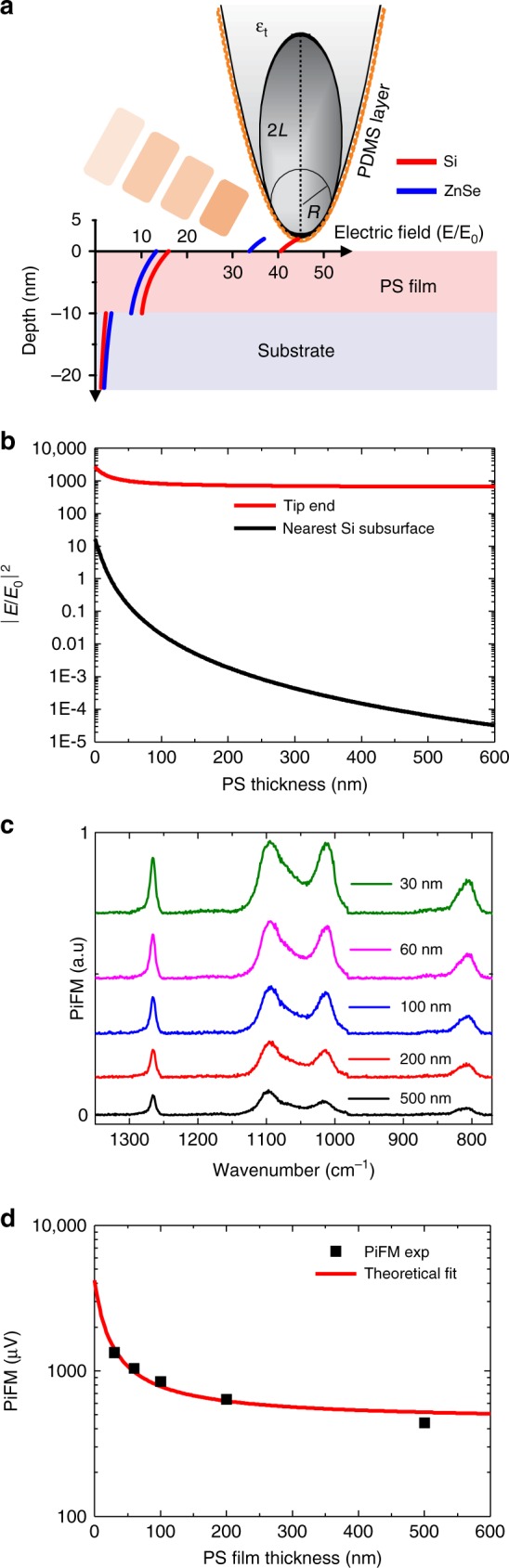
Tip expansion over a layered sample. **a** Electric field distributions along the depth direction from the Au-coated tip end to the 10 nm polystyrene (PS) film on the Si wafer (red solid line) and on the ZnSe window (blue solid line). **b** Normalized field intensities as a function of the PS thickness calculated at the Au-coated tip end (red solid line) and at the Si subsurface (black solid line). **c** Measured tip expansion spectra with respect to polystyrene film thickness (offset for clarity). **d** Comparison between experiment and theory for tip expansion as a function of PS thickness at the molecular vibrational peak (1268 cm^−1^) of the PDMS

The tip expansion is more sensitive to the substructure of the sample than the sample expansion. The electric field distribution for a layered system of 10 nm polystyrene (PS) film on a Si (or ZnSe) substrate is analytically calculated by implementing the finite dipole method, as plotted in Fig. 3a. The red and blue solid lines show data for the Si substrate and ZnSe substrate, respectively. The field distribution normalized by the incident field *E*_0_ shows a monotonic decrease away from the end of the tip followed by a discontinuous drop at the surface of each layer. The degree of the sudden drop depends on the refractive index of each layer. Since the field intensity near the tip end depends more sensitively on the substrate materials, Si or ZnSe substrate in the present case, than does the field inside the substrates, the tip expansion probes the substructure better than does the sample expansion. The normalized field intensity, |*E/E*_0_|^2^, inside the substrate is more rapidly decreased with respect to the PS thickness than at the tip end. The calculated |*E/E*_0_|^2^ at the tip end (red) and just underneath the Si surface (black) is plotted as a function of PS film thickness in Fig. 3b. The calculation details are given in Section 1 of the Supporting Information. The |*E/E*_0_|^2 ^at the tip end is gradually decreased and never reaches zero with respect to the PS thickness. However, the normalized field intensity inside the substrate is approximately two orders of magnitude smaller than at the tip end and is rapidly decreased being close to zero for large PS film thickness.

Figure 3c plots the measured tip-expansion force spectra for the PDMS contaminant with respect to the PS thickness with offsets for clarity. In the measured spectral range, no significant absorption for the PS film and Si substrate is observed, unlike the results obtained for the PDMS. The refractive index of PS (*n*_PS_ ~ 1.59 at 1268 cm^−1^)^27^ is smaller than that of Si (*n*_Si_  ~  3.42 at 1268 cm^−1^)^19^. The PiFM force spectra show a monotonic decrease as the PS film thickness increases because the field enhancement decreases with respect to the PS thickness, as shown in Fig. 3b. This leads to a decreased local absorption on the PDMS at the tip end. By gathering the peak values at 1268 cm^−1^, the change in PiFM signal due to the tip expansion is plotted as a function of the PS thickness along with the calculation result in Fig. 3d. The experimental results correspond remarkably well to the theoretically expected tip expansion given by Eq. (S8). Note that our noise level is ~10 μV, which is far below the measured PiFM signal shown in Fig. 3d.

The enhanced sensitivity due to the tip expansion enables one to characterize the substructure of complex heterogeneous nano/biomaterials, such as polyacrylonitrile-nanocrystalline cellulose (PAN-NCC) nanofiber, which shows a small difference in the refractive indices for the different compositions (*n*_PAN_ ~ 1.32^28^, *n*_NCC_ ~ 1.39^29^ at 1268 cm^−1^). It is well known that polyacrylonitrile (PAN)-based carbon nanofibers are low-cost materials with good environmental sustainability, high efficiency and low energy consumption^30–32^. Compositing nanocrystalline cellulose (NCC) into PAN nanofibers enhances the tensile strength and elastic modulus of the fibers, leading to an enhanced thermomechanical performance. The density and distribution of the NCCs in the PAN are crucial factors that determine the quality of the nanofibers. It is known that the solvent exchange method enables one to distribute the NCCs more uniformly inside the PAN compared to the use of a simple mixing method due to the avoidance of NCC precipitation in the organic solvent^32^. However, interestingly, the tensile strength is actually decreased while the elastic modulus is increased by the solvent exchange method. This finding might be related to the alignment of the NCCs inside the PAN nanofiber; however, so far, it has been very challenging to visualize the substructure of the PAN-NCC nanofiber because they are organic chemicals with only a small difference in refractive indices.

**Fig. 4:**
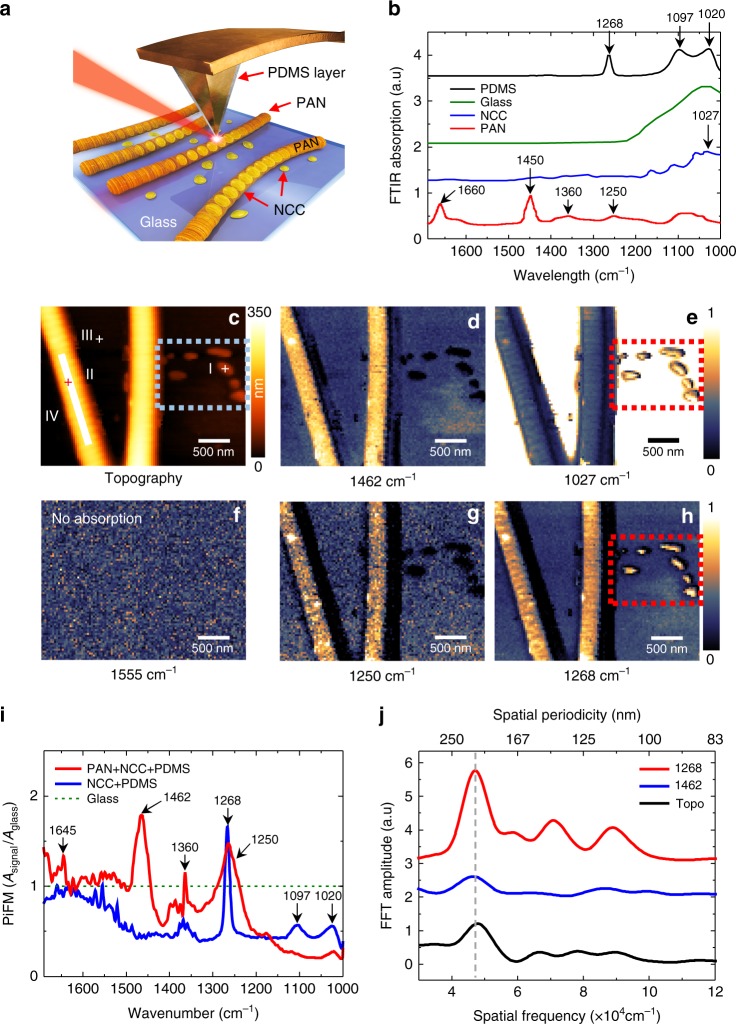
Hyperspectral PiFM images and spectra for PAN-NCC nanofiber in the mid-IR. **a** Sketch of the PAN-NCC nanofibers. **b** FTIR spectra for bulk PAN, NCC, glass, and PDMS obtained from the library. **c** Topography of the PAN-NCC nanofibers. **d**–**h** PiFM images **d** at the PAN absorption resonance (1462 cm^−1^), **e** near the NCC absorption resonance (1027 cm^−1^), **f** away from the absorption resonance (1555 cm^−1^) of any material, **g** at the PAN absorption resonance (1250 cm^−1^), **h** at the PDMS absorption resonance (1268 cm^−1^), which corresponds to a local refractive index contrast for the PAN-NCC nanofiber. **i** Measured PiFM spectra convolved by the tip expansion at position (II) for the PAN-NCC (red solid line) and at position (I) for the bare NCC (blue solid line) in the topography. The data are normalized by the glass spectrum at position (III) in the topography. **j** Spatial frequency plots for three images measured at the line-cut (IV) shown in Fig. 4c

The PiFM successfully visualizes the NCCs deposited inside the PAN nanofiber with nanoscale resolution, as shown in Fig. 4. The PAN-NCC structure is sketched in Fig. 4a. The PAN (11%)-NCC (3%) in *N*,*N*-dimethylformamide (DMF) solution is used to produce electrospun nanofibers, wherein the PAN polymer eventually surrounds the NCC grains^32^. We used the solvent exchange method to mix the NCC into the PAN-DMF solution, which prevents the precipitation of NCC to maintain a uniform distribution in the solution. Figure 4b presents the FTIR absorption spectra for each material (PAN^32^, NCC^32^, glass^33^, and PDMS^20^) as obtained from the library data (shown with offsets for clarity). The two strands of PAN-NCC nanofibers on the glass substrate show strong thermal expansion at 1462 cm^−1^ (CH group vibrations of different modes for the PAN fiber) in Fig. 4d, where the bare NCC debris is not visible. In Fig. 4e, the overall contrast is reversed because of the strong glass absorption in the range 1000–1200 cm^−1^. The bare NCC debris in the red dash box show a stronger thermal expansion signal compared to the NCCs inside the PAN fiber at 1027 cm^−1^ (C–OH deformation mode of the NCC). This is because the field on the PAN-NCC nanofiber should penetrate the PAN to reach the NCCs, while the bare NCC debris directly absorb the field. Note that the PDMS on the tip also shows a strong absorption near the NCC resonance such that the NCC grains might show a slightly enhanced contrast due to the tip expansion contrast. The PiFM force spectra in Fig. 4i clearly show how the PDMS on the tip interferes with the sample spectrum. Normalizing the measured spectra on I (bare NCCs) and II (PAN-NCC) with the glass signal on III in Fig. 4c, one can see that the sample expansion spectra (bare NCC and PAN-NCC) are convolved with the tip expansion (PDMS). Since the glass substrate shows a strong broadband absorption below 1200 cm^−1^, the peaks for the PDMS at 1020 and 1097 cm^−1^ are below the glass substrate signal, as expected. Above 1200 cm^−1^, the main chemical bonds for PAN are well manifested by the representative peaks at 1250, 1360, 1462 and 1640 cm^−1^. The tip expansion is also evident with a sharp peak observed at 1268 cm^−1^. Thus, it is not surprising that there is no signal at 1555 cm^−1^ in Fig. 4f where all the materials (PAN, NCC, glass, and PDMS) are out of resonance, which also implies that the signal due to the dipole force between the tip and the sample is below the noise level.

As shown in Fig. 3, the refractive index contrast between NCC and PAN can be greatly enhanced through tip expansion and clearly visualized at 1268 cm^−1^, where no significant absorption is observed due to the sample and the substrate, except for the PDMS on the tip. In this case, the PiFM image represents the local refractive index map of the sample. Since the peak at 1268 cm^−1^ is a very sharp resonance line due to the Si-CH_3_ deformation mode in the PDMS^20^, the refractive index contrast at 1268 cm^−1^ in Fig. 4h is sharply distinguished from the broad absorption for PAN near 1250 cm^−1^, where the bare NCC debris are not visible in Fig. 4g. Even though the refractive indices for PAN and NCC are comparable to each other, one can clearly distinguish the NCC grains inside the PAN nanofiber in Fig. 4h because of the significantly different field enhancement at the tip end. The NCCs deposited in the PAN nanofiber show a signal level that is comparable to that of the bare NCC debris in the red dashed box, which is in contrast to the PiFM image at 1027 cm^−1^ shown in Fig. 4e. This occurs because the PiFM contrast in Fig. 4h solely depends on the refractive index, rather than the resonant absorption of the sample. The NCCs are expected to be deposited inside the PAN approximately 40 nm below the surface with an effective local refractive index of approximately 1.35 on the deposited NCC, which is determined by the calibration of the measured signal [Section 3 of Supplementary Information]. Through the line-cut on the PAN-NCC strand designated by the white solid line (IV) in Fig. 4c, the alignment of the NCC grains inside the PAN-NCC fiber is clearly manifested. The Fourier transform for the line-cut profile is shown in Fig. 4j. The main spatial frequency of the NCC arrangement is 4.7 × 10^4^ cm^−1^, which corresponds to a spacing of 213 nm. The periodicity is slightly distinct in topography (black solid line), while it is barely discernable at 1462 cm^−1^ (blue solid line); it is the most apparent at 1268 cm^−1^ (red solid line). This result clearly demonstrates the substructure imaging capability of the PDMS-coated tip in PiFM.

The reported tensile strength and elastic modulus for the bare PAN nanofiber and PAN-NCC, which is prepared by using the solvent exchange method, is 17.65 MPa (13.52 MPa) and 0.43 GPa (0.64 GPa), respectively^32^. The periodic alignment of the uniform NCCs may decrease the tensile strength of the PAN nanofiber because the small volume of the PAN polymer between the adjacent NCCs may be easily broken up against external tension. Conversely, the random size and orientation of the NCCs may increase the tensile strength of the nanofiber. The tensile strength of the PAN-NCC fiber prepared using a simple mix method, which leads to a random size and orientation for the NCCs due to precipitation, is reported to lie in the range of 18.63–23.62 MPa^32^, which is the highest value obtained compared to samples prepared using the other methods, as expected. Note that the elastic modulus for the sample prepared using the simple mix method (0.34–0.61 GPa) is slightly smaller than that of the sample prepared by the solvent exchange method (0.64 GPa). Since the elastic modulus depends on the quantity of the deposited NCC in the PAN nanofiber, which may be decreased by random distribution, the hardness of the nanofiber is further increased by use of the solvent exchange method.

## Discussion

We demonstrate that a tiny amount of PDMS on a commercial tip can generate a photoinduced force via tip-enhanced thermal expansion in PiFM, which can be considered a natural functionalization and utilized as a sensitive imaging probe for mapping the local refractive index of a sample with enhanced contrast. Due to its relatively high absorption and high thermal expansion coefficients, the PDMS exhibits a large thermal expansion due to absorption of the field at the tip end, which is based on the refractive index of the sample. Consequently, a large thermal expansion force is generated via the van der Waals interaction, which eventually results in enhanced refractive index contrast for PiFM imaging. The enhanced sensitivity for the refractive index contrast is very useful for characterizing the substructure of a heterogeneous nanobiosample such as a PAN-NCC nanofiber. By visualizing the alignment of the NCC inside the PAN fiber, one can address the issue of the tensile strength of PAN-NCC fibers fabricated by several different mixing methods. This work can be generalized by switching the PDMS on the tip with other functionalizing materials, which show an absorption resonance at the required optical frequency. Based on the present study, our understanding is that it is possible to provide enhanced sensitivity for substructure mapping of organic materials and biomaterials, as well as local field mapping of photonic devices, such as surface polaritons on semiconductor, metal, and van der Waals materials using low illumination power.

## Materials and methods

### PiFM measurements

A VistaScope microscope from Molecular Vista Inc. is used for the mid-IR experiment, which is coupled to a Laser Tune QCL laser system from Block Engineering with a wavenumber resolution of 0.5 cm^−1^ and a tuning range from 800 to 1800 cm^−1^. The microscope is operated in tapping mode with NCH-Au 300 kHz noncontact cantilevers from Nanosensors. Typically, the resonance frequency, quality factor, and stiffness for the fundamental mode is ~300 kHz, with a second resonance of ~1.88 MHz. The 30 ns pulsed beam is modulated by tuning its repetition rate to the difference frequency of the eigenmodes of the cantilever as *f*_m_ = *f*_2_ − f_1_ = 1.58 MHz. The laser beam is side-illuminated onto the sample with an angle of 30° using a parabolic mirror whose numerical aperture (NA) is ~0.4. The average illumination power is ~5 mW and the focus is maintained for all the different tips by scanning the shape of the focal spot with a piezoelectric parabolic mirror. The measured focal spot is optimized with a size of 2.5 *λ* for the long axis and 1.5 *λ* for the short axis, where *λ* = 9.1 μm (1100 cm^−1^). The focal spot images are plotted with respect to the different tip materials on ZnSe in Section 4 of Supplementary Information. The tip locates at the center of the spot (red circle dot) to obtain the spectrum.

The minimum detectable force for a cantilever is derived from the thermal noise of the *i*th eigenmode of the cantilever, which is given as $N_i = \sqrt {4K_{\mathrm B}TBQ_i/\omega _ik_i}$, where *k*_*i*_, *Q*_*i*_, and *ω*_*i*_ are the stiffness, quality factor, and resonance frequency of the *i*th eigenmode of the cantilever, respectively, and *K*_B_ is the Boltzmann constant, *B* the system bandwidth and *T* the absolute temperature. For a typical noncontact cantilever, *ω*_1_ = 2*π* × 300 kHz, *Q*_1_ = 500, *k*_1_ = 37 N m^−1^, *ω*_2_ = 2*π* × 18,800 kHz, *Q*_2_ = 800 and *k*_2_ = 1454 N m^−1^, which gives a thermal noise of *N*_1_ ≈ 1.11 pm and *N*_2_ ≈ 0.09 pm, where *T* = 300 K and *B* = 10 Hz, for the fundamental and second eigenmodes, respectively. The minimum detectable forces, $F_{{\mathrm{min}}_i} = k_iN_i$, are ~0.08 and 0.16 pN for the fundamental and second eigenmodes, respectively.

### PS homopolymers on a Si substrate

The polystyrene film is prepared by spin-coating the homopolymer of PS onto silicon substrates, which is purchased from Polymer Source Inc., Quebec, H9P2X8 Canada. The PS homopolymer has a molecular weight of Mn = 22.5 kg/mol and Mw/Mn = 1.06, resulting in a film thickness of 60 nm.

### PAN-NCC nanofiber

Polyacrylonitrile powder (Mw = 150,000 g/mol, Scientific Polymer Products, Ontario, NY, USA) was dissolved in *N*,*N*-dimethylformamide (Fisher Scientific, Waltham, MA, USA) to produce a solution of 11% PAN by weight. The 3% NCC used in this study was obtained from sulfuric acid hydrolysis of commercially available microcrystalline cellulose (MCC) (Avicel PH-101, Fluka). The 64 wt% MCC/H_2_SO_4_ suspension was hydrolyzed at 45 °C for 2 h with cold water then added to stop the hydrolysis reaction. Then, the suspension was treated by washing and centrifugation. The collected turbid solution was later dialyzed and lyophilized to obtain dried NCC. The solution was then transferred to a plastic syringe and fit to a Nanofiber Electrospinning Unit (NEU–Kato Tech, Japan). The electrospinning unit was operated using the following parameters: applied voltage of 14.6 kV; distance between the needle tip and the collector plate of 10 cm; The syringe pump speed was varied between 0.05 and 1 mL h^−1^ to maintain a clear and visible Taylor cone.

## Electronic supplementary material

Supplementary information
