# Supplementary material for: Substructure imaging of heterogeneous nanomaterials with enhanced refractive index contrast by using a functionalized tip in photoinduced force microscopy
Source: Light Sci Appl. 2018 Oct 10;7:73. doi: 10.1038/s41377-018-0069-y (PMC6177416; doi:10.1038/s41377-018-0069-y)
Supplement: Supplementary file 1 — Supplementary information [file 41377_2018_69_MOESM1_ESM.pdf]

Supplementary Information for

substructure imaging of heterogeneous nanomaterials in the mid-IR range with enhanced refractive index contrast by using a functionalized tip in photoinduced force microscopy

Junghoon Jahng[1], Heejae Yang[2] and Eun Seong Lee[1*]

[1]Center for Nanocharacterization, Korea Research Institute of Standards and Science (KRISS), Daejeon 34113, Republic of Korea

[2]Department of Materials Engineering, Advanced Fibrous Materials Laboratory, University of British Columbia, Vancouver, BC V6T 1Z4, Canada

[*]Corresponding author. Email: eslee@kriss.re.kr (E. S. L.);

S1. Electric potential and field distribution in the layered system.

When a clean sharp metal tip is illuminated on a layered system, the field enhancement near the tip is boosted by the multiple-scattering process between the tip and the sample [1]. The process is rigorously described by using the Green function approach. If we focus on the near-field contribution, it can be further simplified by using the image-dipole method. Hauer *et al.* [2] successfully modeled the multiple-scattering effect in the electrostatic condition by implementing the ellipsoidal finite dipole-image dipole model in the layered system.

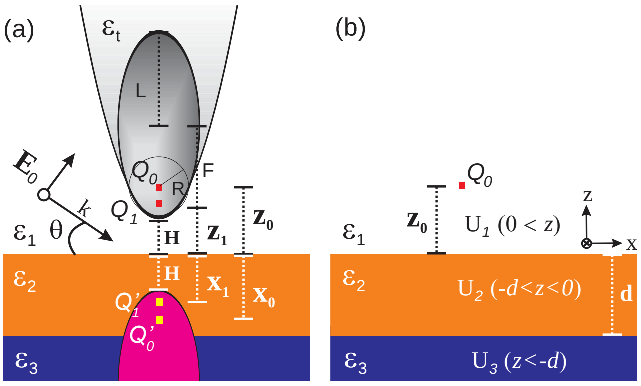

Figure S1 Schematic diagram of the image charges with finite dipole method. (**a**) Dipole-image dipole interaction based on the ellipsoidal finite dipole model. The induced charge on the tip due to the incident field is Q_0 which is positioned at Z_0. The induced charge due to the multiple reflection process with the sample is Q_1 positioned at Z_1. The image charge of them is positioned at X_0 and X_1 in the layered sample. The tip is modeled as an ellipsoid with length of $2L$. The plane wave light is illuminated to the sample with angle of θ. The H is the gap distance from the tip end to the sample surface. (**b**) Potential responses of a charge Q_0 in the distance z_0 above a flat layered sample.

In this model, the potential response U of the sample to the tip can be calculated by considering the two induced charges on the tip and the other two image charges in the sample. The induced charge on the tip due to the incident field is Q_0 which is positioned at Z_0. The induced charge due to the multiple reflection process with the sample is Q_1 positioned at Z_1. The image charge of them is positioned at X_0 and X_1 in the layered sample. By considering the boundary conditions, the potential above the sample is given as [3]

$$U_1 = -\frac{Q_i}{4\pi\varepsilon_0}(\Phi_0 + \Phi_1),$$
$$\Phi_0 = \int_0^\infty e^{-k|z_i - z|} J_0(kr)dk,$$
$$\Phi_1 = \int_0^\infty A(k) e^{k(z_0 - z)} J_0(kr)dk \quad (0<z<z_0). \quad \text{(S1)}$$

The potential inside the sample is given as

$$U_2 = -\frac{Q_i}{4\pi\varepsilon_0}(\Phi_2 + \Phi_3),$$
$$\Phi_2 = \int_0^\infty B(k) e^{-k(z_i - z)} J_0(kr)dk,$$
$$\Phi_3 = \int_0^\infty C(k) e^{k(z_i - z)} J_0(kr)dk \quad (-d<z<0). \quad \text{(S2)}$$

The potential below the sample (inside substrate) is given as

$$U_3 = -\frac{Q_i}{4\pi\varepsilon_0}\Phi_4,$$

$$\Phi_4 = \int_0^{\infty} D(k) e^{-k(z_i - z)} J_0(kr) dk, \quad (z<-d) \quad \text{(S3)}$$

where *i*=*0*,1 with $Q_0 = 4\pi\varepsilon_0 R^2 \frac{\frac{2F(L)}{L(a)} + \mathrm{Log}[\frac{L-F}{L+F}]}{\frac{2F(L-\varepsilon_t a)}{La(\varepsilon_t - 1)} - \mathrm{Log}[\frac{L-F}{L+F}]} E_0$ and $Q_1 = \frac{\beta_{X_0} f_0}{1-\beta_{X_1} f_1} Q_0$. The ε_0 is the permittivity of the vacuum, $\beta_{X_i} = -\frac{\Phi_1(z_i)^2}{\Phi'_1(z_i)}|_{z=0}$, $f_i = (g - \frac{R+2H+z_i}{2L}) \frac{\ln\frac{4L}{R+4H+2z_i}}{\ln\frac{4L}{R}}$, and $A(k) = \frac{\beta_{12}+\beta_{23}e^{-2kd}}{1-\beta_{21}\beta_{23}e^{-2kd}} e^{-2kz_0}$, $B(k) = \frac{2}{\epsilon_2+1} \frac{1}{1-\beta_{21}\beta_{23}e^{-2kd}}$, $C(k) = \frac{2}{\epsilon_2+1} \frac{\beta 23}{1-\beta_{21}\beta_{23}e^{-2kd}} e^{-2k(d+z_0)}$ and $D(k) = \frac{2}{\epsilon_2+1} \frac{1+\beta_{23}}{1-\beta_{21}\beta_{23}e^{-2kd}}$. The β_{nm} is the electrostatic reflection factor for the layered system, given as $\beta_{nm} = \frac{\varepsilon_n - \varepsilon_m}{\varepsilon_n + \varepsilon_m}$ for *n*, *m*=1,2,3. The *g* is the empirical geometric factor due to the tip shape. For typical PiFM (or s-SNOM) tip geometries, $|g|$ = 0.7±0.1 [4]. The total potential response is the sum of the potentials given as $U_n = U_n(Q_0, z_0) + U_n(Q_1, z_1)$ where n = 1,2,3, and the electric field is given by differentiating the potential with respect to the z-axis, $E_n = -\frac{\partial U_n}{\partial z}$, in the regions. Note that this ellipsoidal geometry doesn't show any resonant antenna effect in the mid-IR, which is related to the tip's geometry [5].

The electrostatic potential and the normalized field distribution inside and outside of the 10 nm PS film on Si substrate and on are plotted in Fig. S2a and S2b where the gap distance H is fixed by 2 nm, by implementing the library value of the bulk polystyrene [6], gold [7] and Si substrate [8]. The simulation parameters are R = 30 nm, L = 450 nm, τ_p = 30 ns, H = 2 nm, θ = 30 degree and the ν_0 = 1268 cm^{-1}. Let's assume the incident electric field as E_0 = 10^6 V m^{-1} which covers the experimental parameters of the I_0 = 5 mW (incident power), A = π(10 μm)^2 (focal area), f = 1.6 MHz (repetition rate) and τ_p = 30 ns (pulse width). The tip end is located at z = 2 nm.

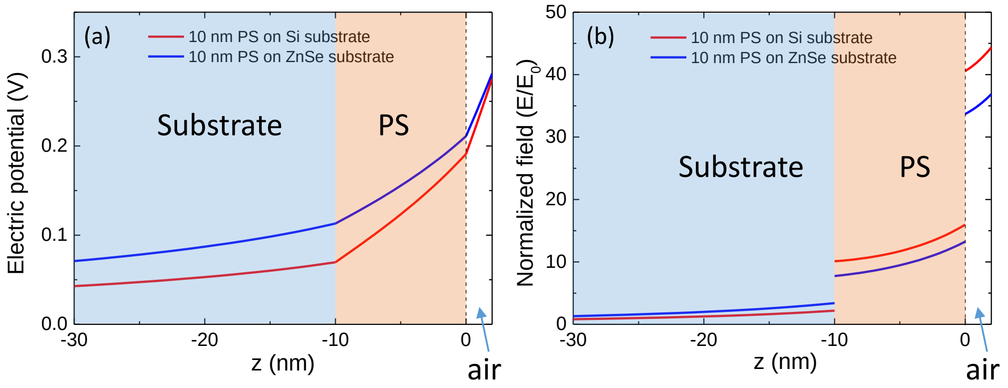

Figure S2 Electric potential and field distribution on the polystyrene film over Si substrate. (**a**) Calculated electrostatic potential and (**b**) normalized field distribution by E_0 on the PS film over Si substrate (red solid line) and ZnSe substrate (blue solid line) from tip end to substrate at the resonance of the PDMS (1268 cm^{-1}). The sample surface is located at z = 0 and the tip end is located at z = 2 nm.

S2. Calculation of the tip-enhanced photo-thermal expansion force for the PDMS contamination via van der Waals interaction in PiFM

Let us next calculate the tip-enhanced photo-thermal expansion force of the contaminated PDMS on the tip over the PS film on the Si substrate. The photo-thermal expansion force is the result of several causal processes: First, there is an energy exchange with the light field, which scales with the optical absorption coefficient and results in a temperature rise (ΔT ~ P_{abs}). Second, the accumulated heat diffuses to deform the sample to induce a thermal expansion (ΔL ~ ΔT). Third, the thermal expansion changes the tip-sample distance, which introduces a modulation of the tip-sample interaction force (ΔF ~ ΔL). Then the gradient of the modulated force is coupled to the PiFM.

When we assume the PDMS layer is 1 nm [9], it may be ignorable for about the field distribution. In this case, the electric field is approximately calculated for the clean Au tip case. The absorbed power is obtained by integrating $|E|^2$ from the tip end toward the substrate by 1 nm in Fig. S2b. After calculating the electric field distribution, the PDMS will be revisited in the force calculation. By integrating the $|E|^2$ at the tip end and substituting it into $P_{abs} = \int a_{abs} \frac{1}{2} c\varepsilon_0 |E|^2 \, dV_{abs}$ where $a_{abs} \approx \frac{4\pi}{\lambda} \frac{\mathrm{Re}[n]\mathrm{Im}[n]}{\sqrt{\mathrm{Re}[n]^2 - \mathrm{Im}[n]^2}}$ [10,11], then one can obtain the absorption coefficient of the PDMS to 91.2 x 10^4 m^{-1} at the vibrational resonance of 1097 cm^{-1}. If we assume that the thermal expansion is proportional to the temperature change, the calculation of the thermal expansion is much simplified by regarding the maximum temperature change, given as [11,12]:

$$\Delta T_{max} \approx \frac{P_{abs}}{\rho C V_{heat}} \tau_{rel} \qquad (\tau_{rel} < \tau_p) \qquad \text{(S4)}$$

$$\Delta T_{max} \approx \frac{P_{abs}}{\rho C V_{heat}} \tau_p \qquad (\tau_{rel} > \tau_p) \qquad \text{(S5)}$$

where V_{heat} is the heated volume related to the thermal wavelength [13], given as $l_p \approx \sqrt{D\tau_p}$ where $D \approx \frac{1}{1.13} \frac{\pi^2}{4} \frac{\kappa_{eff}}{\rho C}$ which is the thermal diffusivity [14]. The tip-enhanced thermal expansion is given as:

$$\Delta L_{max} \approx \sigma d \Delta T_{max} \qquad (S6)$$

where σ is the linear thermal expansion coefficient. The relaxation time is given as $\tau_{rel} \approx d^2/D$. The linear thermal expansion coefficient, density, heat capacity and thermal conductivity of the PDMS are 907×10^{-6} K^{-1}, 965 kg/m^3, 1460 J/kg.K and 0.15 W/m.K, respectively. The κ_{eff} is the effective thermal conductivity which serially connects the sample material to the thermal bath given as $\frac{1}{\kappa_{eff}} \approx \frac{1}{\kappa_{sample}} + \frac{h}{d}$ where the h is the interfacial thermal resistance between the sample and the thermal bath. For the thin films of low thermal conductivity such as PDMS is the interfacial thermal resistance between PDMS and the gold coating become the dominant factor for the heat transfer. Unluckily the interfacial thermal resistance is often unknown which make modelling of the thermalization dynamic difficult, but has the general effect of increasing the relaxation time with more prominent effect the thinner the sample. According to Ref. [14] we assume this effect decreases the k of the sample around by 1/10 for the 1 nm PDMS so that $\kappa_{\mathrm{eff}} \approx \frac{1}{10}\kappa_{\mathrm{PDMS}}$. Then the thermal diffusivity of the PDMS is 2.32 $\times$ 10^{-8} m^2/s and thus the relaxation time is of the PDMS is $\tau_{\mathrm{rel}} \approx \frac{(1nm)^2}{2.31\times10^{-8}m^2/s} \approx 43$ ps. Because the relaxation time is smaller than the pulse duration (τ_p = 30 ns), Eq. (S4) is used for the maximum thermal expansion calculation. In this case the maximum thermal expansion is rewritten as:

$$\Delta L_{\max} \approx \sigma d \Delta T_{\max} \approx \frac{\sigma d P_{abs} \tau_{rel}}{\rho C V_{heat}} \approx \frac{\sigma \tau_{rel}}{\rho C d} \int a_{abs} \frac{1}{2} c \varepsilon_0 |E|^2 \, dz. \qquad (S7)$$

We assume the $V_{heat} \approx A_{heat} d$ and $A_{heat} \approx A_{abs}$ for the PDMS contamination. For the PDMS contaminated tip on the Si substrate with H = 2 nm, the electric field is plotted as in Figure S2b. Because the PDMS thickness is regarded as 1 nm, by integrating the electric field from the tip end by 1 nm in Figure S2b and substituting it into Eq. (S13), then one can obtain the thermal expansion of ~*24* pm at the PDMS vibrational resonance of 1268 cm^{-1}. Finally, the photo-thermal expansion force in tapping mode PiFM is mediated by the modulated tip-sample interaction force which constitutes the attactive van der Waals force and the repulsive DMT contact force. Because the modulated DMT contact force [15] due to the oscillotary thermal expansion is under the noise level (sub pN) due to the extremly small elasticity of the PDMS (0.6 MPa), the modulated van der Waals force and its force gradient contribute to the PiFM signal, given as:

$$\Delta F_{\mathrm{vdW}} \approx -\frac{H_{\mathrm{eff}}R}{12}\left(\frac{1}{(H-\Delta L)^2} - \frac{1}{H^2}\right) \approx -\frac{H_{\mathrm{eff}}R}{6}\frac{1}{H^3}\Delta L \quad (H>r_0) \qquad (S8)$$

$$\Delta \frac{\partial F_{\mathrm{vdW}}}{\partial z} = \frac{H_{\mathrm{eff}}R}{6}\left(\frac{1}{(H-\Delta L)^3}\left(\frac{\partial H}{\partial z} - \frac{\partial(\Delta L(H))}{\partial z}\right) - \frac{1}{H^3}\frac{\partial H}{\partial z}\right) \approx -\frac{H_{\mathrm{eff}}R}{6}\frac{1}{H^3}\frac{\partial(\Delta L)}{\partial H} \quad (H>r_0) \qquad (S9)$$

where the r_0 is the intermolecular distance given as 0.3 nm, H_{eff} is the effective Hamaker constant between tip and sample, R is the tip radius. The Hamaker constant between the PDMS and Si substrate is described as the reduced Hamaker constant which is given as $H_{\mathrm{eff}} = \sqrt{H_{\mathrm{pdms}} \times H_{\mathrm{Si}}}$ = 12.7 x 10^{-20} J where H_{pdms} is 6.3 x 10^{-20} J and H_{Si} is 25.6 x 10^{-20} J. The modulated van der Waals force is around 16 pN for ΔL = 24 pm, R = 30 nm at 1 nm gap distance from the PDMS on the tip end to the Si surface.

S3. Calibration for the effective local index and the depth of the substructure

By comparing the PiFM signal on bare NCC and on PAN-NCC, the effective local refractive index of PAN-NCC structure is able to be calibrated. Because the PiFM signal is proportional to the field-enhancement, one can estimate the effective local index by using the relation of $\frac{\left(\frac{n_{\mathrm{eff}}^2-1}{n_{\mathrm{eff}}^2+1}\right)^2}{\left(\frac{n_{\mathrm{NCC}}^2-1}{n_{\mathrm{NCC}}^2+1}\right)^2} \approx \frac{\mathrm{PiFM\ on\ } n_{\mathrm{eff}}}{\mathrm{PiFM\ on\ } n_{\mathrm{NCC}}}$. According to Hilfiker *et al.* [16], if a polymer size is over 10 nm, the bulk index is still applicable to the nanosystem. The bulk indices for PAN and NCC are known as n_{PAN}=1.32 [17] and n_{NCC}=1.39 [18] at 1268 cm^{-1}. The ratio of the measured PiFM signal on PAN-NCC (B) to the NCC (A) in Fig. S3a is 0.84. From the above relation, the measured effective index of the PAN-NCC is estimated by 1.35.

The normalized electrostatic field distribution at the tip end on the PAN-NCC nanofiber is calculated as a function of PAN thickness in Fig. S3b, where the gap distance between Au coated tip end and the PAN surface is fixed by 2 nm, by implementing the library value of the refractive index of PAN and NCC (n_{PAN}=1.32 [17], n_{NCC}=1.39 [18] at 1268 cm^{-1}). The simulation parameters are R = 30 nm, L = 450 nm, τ_p = 30 ns, H = 2 nm, θ = 30 degree, E_0 = 10^6 V m^{-1} and the ν_0 = 1268 cm^{-1}. The measured PiFM signal ratio of PAN-NCC (B) to the NCC (A) in Fig. S3a is 0.84. The expected PAN thickness is around 40 nm from the calculation curve in Fig. S3b, because that the PiFM signal is proportional to the $|E|^2$ where $\left(\frac{E(d=40\mathrm{nm})}{E(d=0\mathrm{nm})}\right)^2 \approx 0.84$.

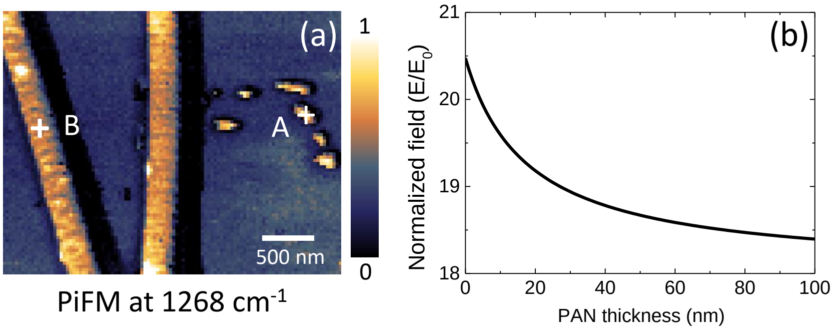

Figure S3 (**a**) PiFM image of the PAN-NCC sample at the resonance of the PDMS (1268 cm^{-1}). (**b**) Calculated field at the tip end

on the PAN-NCC sample with respect to the PAN thickness.

S4. Laser focal spot images with respect to different tip materials on ZnSe

The measured focal spot images are obtained by different tip materials on ZnSe in Figure S4. The scan size is 33 µm x 33 µm and the measured focal spot is optimized with the size of 2.5 λ for long axis and 1.5 λ for short axis where λ = 9.1 µm (1100 cm^{-1}). After mapping the focal spot, we put the tip into the center of the spot (red circle dot) to obtain the spectrum in Figure 2b. We keep the same laser power of ~10 mW with pulse width of 30 ns and tried to maintain the same focus for the three different tips by scanning the shape of the focal spot with the piezo electric parabolic mirror.

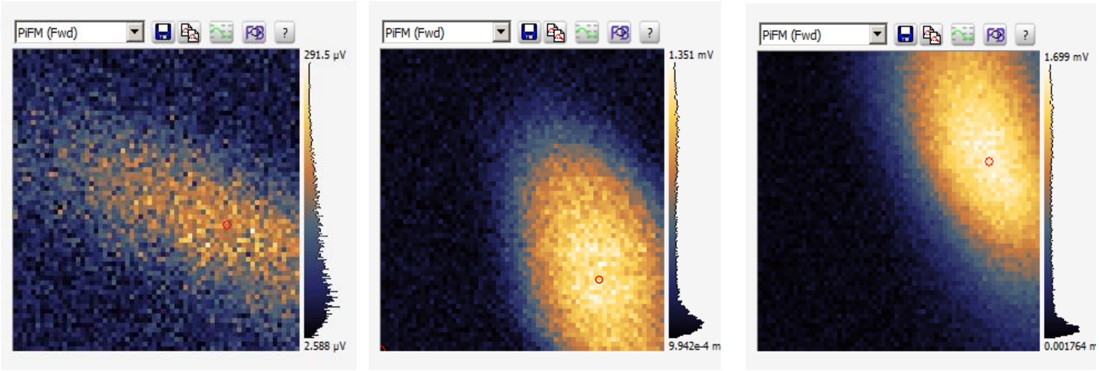

(a) Si tip on ZnSe (b) Au tip on ZnSe (c) PtSi tip on ZnSe

Figure S4 Laser focal spot images with respect to the (a) Si, (b) Au and (c) PtSi tip on ZnSe substrate. The incident wavenumber is 1100 cm^{-1} which corresponds to 9.1 µm.

S5. Refractive index of polymers in mid-IR

The complex index of refraction shows the wavenumber dependent behavior which is generally the dispersive line shape near the molecular resonance. The refractive indices of NCC [18], PAN [17], PDMS [19] and PS [20] in mid-IR range are plotted with respect to wavenumber from the library in Figure S5. The values of n_{PAN} ~ 1.32, n_{NCC} ~ 1.39, n_{PDMS} ~ 1.09 and n_{PS} ~ 1.59 at 1268 cm^{-1} (grey dashed line) are implemented in the main text.

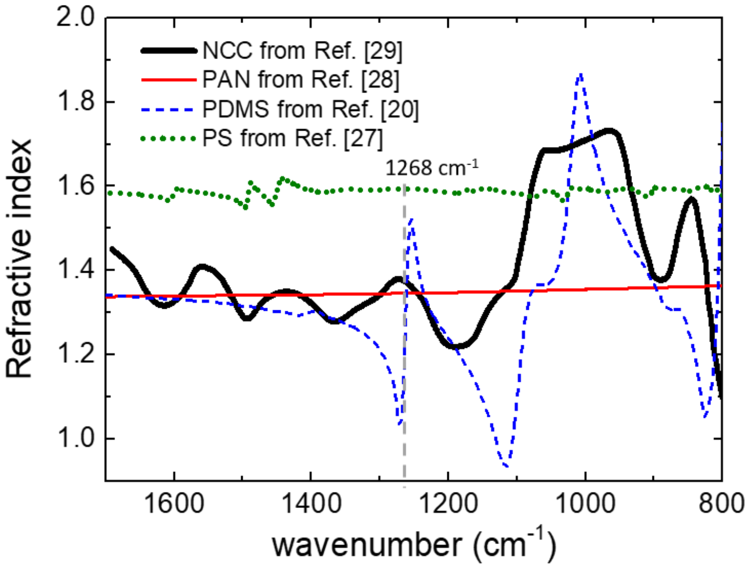

Figure S5 Refractive indices of NCC (black solid line), PAN (red solid line), PDMS (blue dashed line) and PS (green dotted line).

References

1 Ladani, F. T. & Potma, E. O. Dyadic Green's function formalism for photoinduced forces in tip-sample nanojunctions.

Physical Review B **95**, 205440 (2017).
2 Hauer, B., Engelhardt, A. P. & Taubner, T. Quasi-analytical model for scattering infrared near-field microscopy on layered systems. *Optics express* **20**, 13173-13188 (2012).
3 Wang, B. & Woo, C. Atomic force microscopy-induced electric field in ferroelectric thin films. *Journal of applied physics* **94**, 4053-4059 (2003).
4 Cvitkovic, A., Ocelic, N. & Hillenbrand, R. Analytical model for quantitative prediction of material contrasts in scattering-type near-field optical microscopy. *Optics express* **15**, 8550-8565 (2007).
5 Huth, F. *et al.* Resonant antenna probes for tip-enhanced infrared near-field microscopy. *Nano letters* **13**, 1065-1072 (2013).
6 Zolotarev, V., Volchek, B. & Vlasova, E. Optical constants of industrial polymers in the IR region. *Optics and spectroscopy* **101**, 716-723 (2006).
7 Babar, S. & Weaver, J. Optical constants of Cu, Ag, and Au revisited. *Applied Optics* **54**, 477-481 (2015).
8 Chandler-Horowitz, D. & Amirtharaj, P. M. High-accuracy, midinfrared ($450\ cm^{-1} \leqslant \omega \leqslant 4000\ cm^{-1}$) refractive index values of silicon. *Journal of Applied physics* **97**, 123526 (2005).
9 Sirghi, L., Kylian, O., Gilliland, D., Ceccone, G. & Rossi, F. Cleaning and hydrophilization of atomic force microscopy silicon probes. *The Journal of Physical Chemistry B* **110**, 25975-25981 (2006).
10 Jackson, J. D. *Classical electrodynamics*. (John Wiley & Sons, 2012).
11 Dazzi, A., Glotin, F. & Carminati, R. Theory of infrared nanospectroscopy by photothermal induced resonance. *Journal of Applied Physics* **107**, 124519 (2010).
12 Jahng, J., Potma, E. O. & Lee, E. S. Tip-enhanced thermal expansion force for nanoscale chemical imaging and spectroscopy in photo-induced force microscopy. *Analytical Chemistry*, doi:10.1021/acs.analchem.8b02871 (2018).
13 Black, E. D., Grudinin, I. S., Rao, S. R. & Libbrecht, K. G. Enhanced photothermal displacement spectroscopy for thin-film characterization using a Fabry-Perot resonator. *Journal of applied physics* **95**, 7655-7659 (2004).
14 Chae, J. *et al.* Nanophotonic atomic force microscope transducers enable chemical composition and thermal conductivity measurements at the nanoscale. *Nano letters* **17**, 5587-5594 (2017).
15 Lu, F., Jin, M. & Belkin, M. A. Tip-enhanced infrared nanospectroscopy via molecular expansion force detection. *Nature photonics* **8**, 307 (2014).
16 Hilfiker, J. N. *et al.* Determining thickness and refractive index from free-standing ultra-thin polymer films with spectroscopic ellipsometry. *Applied Surface Science* **421**, 508-512 (2017).
17 Tański, T., Matysiak, W. & Hajduk, B. Manufacturing and investigation of physical properties of polyacrylonitrile nanofibre composites with SiO_2, TiO_2 and Bi_2O_3 nanoparticles. *Beilstein journal of nanotechnology* **7**, 1141 (2016).
18 Baillis, D., Coquard, R. & Moura, L. Heat transfer in cellulose-based aerogels: analytical modelling and measurements. *Energy* **84**, 732-744 (2015).
19 Querry, M. Optical constants of minerals and other materials from the millimeter to the ultraviolet. (CHEMICAL RESEARCH DEVELOPMENT AND ENGINEERING CENTER ABERDEEN PROVING GROUNDMD, 1987).
20 Jitian, S. Determination of optical constants of polystyrene films from ir reflection-absorption spectra. *Analele Universităţii Eftimie Murgu Reşiţa. Fascicula de Inginerie* **18**, 41-48 (2011).
